# Sex-specific differences in functional traits and resource acquisition in five cycad species

**DOI:** 10.1093/aobpla/plx013

**Published:** 2017-04-05

**Authors:** Christopher Krieg, James E. Watkins, Sally Chambers, Chad E. Husby

**Affiliations:** 1 Department of Biology, Colgate University, Hamilton, NY 13346, USA; 2 Montgomery Botanical Centre, Miami, FL 33156, USA; 3 Department of Biology, University of Florida, Gainesville, FL 32611, USA; 4 Present address: Fairchild Tropical Botanic Garden, Miami, FL 33156, USA

**Keywords:** Dioecy, evolution, natural isotope abundance, photosynthesis, root symbiosis, trade-offs

## Abstract

Selective pressures acting on plant life histories can drive extreme specialization. One example of such specialization is the evolution of dioecious breeding systems. Evolutionary and ecological theory posits that dioecy may subject male and female individuals to different selective pressures and result in unique sex-mediated adaptive traits related to resource allocation and ecophysiology. Cycads are the earliest diverging lineage of seed plants with strict dioecy, yet we know almost nothing about the ecology and physiology of this group. Especially limited is our understanding of potential sex-specific differences and how such differences may influence species ecology. Here we examine the ecophysiology of male and female cycads to understand better, the role that dioecy plays in this group. We evaluated sex-specific differences in ecophysiological traits and resource acquisition in five species. Specifically, we compared photosynthetic physiology, nitrogen and carbon content, isotope discrimination (δ^15^N and δ^13^C), and stomatal density. In some cycads, (i) males and females have similar investments in leaf nitrogen but females exhibit greater incorporation of nitrogen from nitrogen-fixing soil bacteria, (ii) males display higher photosynthetic capacity but females show greater water-use efficiency, and (iii) males have higher stomatal conductance but similar stomatal density to females. This study is the first to examine the ecophysiological differences that have evolved in the oldest dioecious lineage of seed-bearing plants. Our results show unexpected differences in photosynthetic physiology and highlight the co-evolution with nitrogen fixing soil bacteria as a potential new key player in an old lineage.

## Teaser

The Mesozoic is often referred to as the “age of Cycads.” During this period, dinosaurs roamed vast Cycad forests, yet modern Cycads are a vestige of their Mesozoic glory. Extant species represent the oldest lineage of dioecious seed-bearing plants. This curious phylogenic position is matched by their unusual ecology: most species are extremely rare while others form dense colonies that may play important roles in forest function. In spite of this and quite remarkably, Cycads are the most poorly studied lineage of seed plants and almost nothing is known of their ecophysiology. The study by Krieg et al. is the first of its kind to examine sex-mediated ecophysiology in Cycads. Their results show unexpended differences in photosynthetic physiology and highlight the role that nitrogen fixing soil bacteria may play in Cycad reproduction and ecology. They found that species can vary markedly in nitrogen relations and that plant sex can drive unique leaf physiology. Their study is a cycadological call to arms for plant scientists to refocus efforts on this enigmatic group.

## Introduction

Understanding variation in sexual reproduction and mating systems has long been an area of interest in biology (Darwin 1876, 1877; Stebbins 1950; Bawa 1980; Hurst and Peck 1996; Richards 1986; Peck *et al.* 1998). Nowhere is this more striking than in dioecious plant species. Several hypotheses have been developed to explain the evolution of dioecy in vascular plants, including enhanced genetic diversity, the separation of ecological niches of males and females, and economic theory among others (Case and Barrett 2001; Ross 1982; Charlesworth and Charleworth 1978a,1978b; Bawa 1980; Givnish 1980, 1982; Muenchow 1987). Additionally, many studies have shown sex-specific patterns of resource allocation and growth (Lloyd 1982; Charnov 1982; Givnish 1980; Campbell 2000). Such patterns are often related to the costs of reproduction and the tradeoffs that arise as plants balance reproduction with growth and maintenance (Reznick 1985).

The majority of studies on the physiology of dioecy have demonstrated increased carbon costs in female reproduction. These costs frequently result in net loss of allocation to other processes and incur a greater cost of reproduction in females (Reznick 1985; Ashman 1994; Nicotra 1999a, 1999b; Rocheleau and Houle 2001; Obeso 2002). This is relatively intuitive in flowering plants, where females produce and maintain flowers, fruits, and seeds. Although only 5 % of flowering plants are dioecious, a significant amount of research has been devoted to understanding the evolution and ecology of dioecy in angiosperms (e.g. Obeso 2002). Yet, almost nothing is known of sex-specific patterns of reproduction, and possible evolutionary and ecological consequences in dioecious gymnosperms like the cycads. Such paucity of information is especially surprising given that dioecy in seed plants first appeared ∼300 million years ago in the cycads (Stevenson 1990; Brenner *et al.* 2003; Chaw *et al.* 2005; Friis *et al.* 2006), and the Cycadales are the earliest diverging lineage of vascular plants with complete dioecy (Zhifeng and Thomas 1989; Crane 1988; Mamay 1969; Stevenson 1990, 1992). To date, no bisexual or hermaphroditic cycad has been found in the fossil record (Gorelick and Osborne 2007). With 344 recognized species (Calonje *et al.* 2016), and consistent dioecy among species, cycads offer a unique insight into the evolution and physiology of dioecy in vascular plants.

To date, most work on dioecy in cycads has focused on differences in morphology and leaf production (Newell 1983, 1985; Clark and Clark 1988; Tang 1990). In all genera of cycads (except *Cycas*) males and females produce compact strobili (frequently referred to as cones) composed of either megasporophylls or (and on separate plants) microsporophylls. In *Cycas*, megasporophylls form in the same phyllotactic spiral as the leaves, and are loosely aggregated and do not form tightly compacted strobili. What little work that has been done on cycad ecology suggests that there may be sex-mediated differences in several species. For example, Tang (1990) examined natural populations of *Zamia pumila* and found most populations were male biased and that male reproductive cycles were shorter, and male strobili were smaller, compared to females. Newell (1985) and Tang (1990) found that leaf number and morphology differed between males and females of *Zamia pumila* with males producing fewer leaves. While both of these studies demonstrate sex-mediated ecological differences neither overtly tested the costs of reproduction. Clark and Clark (1988) were one of the first to demonstrate the cost of female reproduction in *Zamia skinneri*, where females (but not males) experienced a decrease in leaf production for two years after reproduction. Further, Ornduff (1987) reported the effects of environmental conditions on the frequency of reproduction and sex-ratio in cycad populations, and found that populations in higher light conditions produced more females than populations in lower light conditions. Marler and Dongol (2011) found evidence that megasporophyll and microsporophyll development in *Cycas micronesica* was canalised and their size was not affected by habitat or season. This suggests that whereas environment may influence sex-ratios and coning frequency, the size and morphology of cycad strobili may be consistent across environments.

Given that habitat and environment have been found to influence sex-ratios and reproduction in cycads, it surprising that little attention has been paid to potential mechanisms involving roots and belowground factors between male and female cycads. All cycad species form root associations with nitrogen-fixing cyanobacteria (e.g. Rai *et al.* 2000; Costa and Lindblad 2002). However, these symbiotic relationships are not well understood in cycads, and no studies have investigated if these associations result in different nitrogen benefits between males and females.

To our knowledge, only four publications have examined cycad photosynthetic physiology (Marler and Willis 1997; Brodribb *et al.* 2007; Álvarez-Yépiz *et al.* 2014; Zhang *et al.* 2015) and none of these studies examine photosynthetic physiology in the context of dioecy. Thus we know little about the physiological differences between the sexes in cycads. Here, we present the first study to investigate sex-specific differences in ecophysiological traits of dioecious cycads. We predict that females will exhibit higher photosynthetic rates to compensate for higher reproductive costs, and that related measures of respiration and stomatal conductance will also be higher in females. We do not predict any differences in leaf nitrogen content or biologically fixed nitrogen between males and females because we do not expect investment in root functions, including nutrient uptake and symbioses, to be affected by sex.

## Methods

All plant species were grown at Montgomery Botanical Centre (MBC), Miami, FL, USA. Individuals included in this study were subjected to a common watering-regime of artificial irrigation and rain, and grown in well-drained soil. The climate is subtropical with an average annual temperature of 25.05 °C and annual precipitation of 157.3 cm. During the early summer months, the average temperature is about 30 °C (US Climate Data, usclimatedata.com).

We selected species within an area of 1 km^2^ from the cycad Geographic Collection at MBC. Each species was grown in proximity to form discrete populations. Specifically, we selected five species, *Cycas micronesica* (*n* = 12), *Cycas rumphii* (*n* = 10), *Zamia potoricensis* (*n* = 24), *Zamia erosa* (*n* = 10), and *Zamia standleyi* (*n* = 12). Males and females were equally represented within species. These species represent key phylogenetic and morphological differences among extant cycad species, with *Cycas* sister to the rest of cycads and *Zamia* having diverged from sister cycad taxa more recently (Nagalingum *et al.* 2011). Additionally, we chose these genera because of significant morphological differences in growth form and reproduction in an effort to maximize morphological variation in our sampling to bolster the generalizability of the findings. For example, the *Zamia* species have subterranean stems that produce cone-like strobili in males and females, whereas the *Cycas* species have large arborescent stems and females produce large un-aggregated megasporphylls. Only reproductively mature individuals were included in this study.

### Gas exchange

In June 2014, we recorded photosynthetic parameters on mature, fully expanded leaves approximately 1/3 of the distance from the apex to the oldest leaflet. We measured maximum photosynthetic rate (*A*_max_), stomatal conductance (*g*_max_) and respiration (*R*_max_), using a Li-Cor LI-6400 portable photosynthesis system (Li-Cor Biosciences Inc., Lincoln, NE, USA). Based on preliminary light response curve data, all taxa reached maximum values at a light level of 1500 µmol m^−2^ s^−1^ with a flow rate of 400 µmol s^−1^ and the CO_**2**_ mixer set to 400 µmol m^−2^ s^−1^. These chamber conditions were then used throughout the study. When compared to ambient temperature and humidity, controlling for leaf or block temperature to maintain the average ambient temperature for the time of year did not alter photosynthetic values; therefore, leaves were exposed to ambient temperature and humidity. Average chamber VPD was 1.5 ± 0.46 kPa, average leaf temperature (*T*_leaf_) was 31.8 ± 2 °C, with an average relative humidity (RH) of 65 %. All measurements were taken between 900 and 1300 h. Respiration rates were recorded by turning off the internal light source and waiting for rates to stabilize.

### Leaf nutrient content

Leaf pinna samples were analysed for nitrogen and carbon content (%N and %C) and isotope discrimination (δ^15^N and δ^13^C) using mass spectrometry facilities in the Department of Biology at Colgate University.

Leaf samples were oven dried at 60 °C and ground into a powder using a Wiley Mill (Thomas Scientific Model 3383-L10) and were then weighed and rolled into tin capsules (5 × 9 mm Costech pressed tin capsules). Samples were then run through an elemental analyser, Costech Instruments Elemental Combustion System (Costech Analytical Technologies Inc. Valencia, CA, USA) in tandem with a Delta Plus Advantage Stable Isotope Mass Spectrometer (Thermo Fisher Scientific Inc. Waltham, MA, USA) at Colgate University. Leaf isotope values are expressed in delta notation (‰) relative to the standard PDB (Pee Dee Belemnite) and air, for C and N, respectively.

### Stomata measurements

For each individual, four leaf disks were cut from the leaflet directly adjacent and above to those used in gas-exchange measurements, and then were mounted in glycerol. Samples were examined with an Olympus BX-40 microscope (Olympus America Inc. Melville, NY, USA). Stomatal densities were measured using a ×40 lens.

### Statistical methods

Gas exchange, leaf nutrient content, and morphological measurement data were analysed separately as a function of cycad sex, species, and the subsequent sex × species interaction using general linear models (PROC GLM in SAS v. 9.3; SAS Institute 2011). In each model, species and sex were modelled as fixed effects, and sexes belonging to the same species were directly compared using contrast statements.

## Results

We found several patterns of ecophysiological differences between sexes including photosynthetic traits, and resource acquisition patterns via symbiotic associations with nitrogen fixing soil bacteria.

### Gas exchange

**Figure 1:**
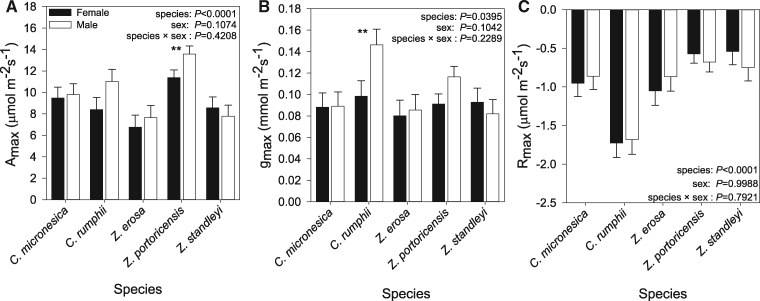
(A) Mean (±1 S.E.) gas-exchange data of photosynthetic maximum (*A*_max_), (B) maximum stomatal conductance (*g*_max_), and (C) maximum respiration (*R*_max_). White bars represent males, and black bars represent females. Double asterisks indicate a significant difference (*P* < 0.05); error bars represent standard error of the mean.

Overall, species differed significantly with respect to maximum photosynthetic rates [species: *F*_4,57_ = 10.37, *P* < 0.0001], with *Z. portoricensis* exhibiting higher *A*_max_ on average relative to the remaining four species [*Z. portoricensis*: 12.473 μmol m^−2^ s^−1^]. Sex and the interaction between sex and species were not significant [sex: *F*_1,57_ = 2.68, *P*  = 0.1074; sex × species: *F*_4,57_ = 0.99, *P*  = 0.4208]. While not significant, males exhibited a slightly greater A_max_ than females on average [male: 9.963 μmol m^−2^ s^−1^, female: 8.911 μmol m^−2^ s^−1^]. Results of the model contrast statements indicate that male and female A_max_ differed significantly for *Z. portoricensis* [*F*_1,57_ = 4.40, *P*  = 0.0404]. For this species, males exhibited a greater A_max_ than females (Fig. 1A).

Maximum stomatal conductance (*g*_max_) differed significantly among species [*F*_4,57_ = 2.70, *P*  = 0.0395], but was not significantly between sexes [*F*_1,57_ = 2.73, *P*  = 0.1042] nor the subsequent sex × species interaction [*F*_4,57_ = 1.45, *P*  = 0.2289]. For this metric, *C. rumphii* and *Z. portoricensis* exhibit the two highest *g*_max_ values on average relative to the remaining three species [*Z. portoricensis*: 0.104 mmol m^−2^ s^−1^, *C. rumphii:* 0.122 mmol m^−2^ s^−1^]. Although overall *g*_max_ of males and females did not differ significantly, model contrast statements indicate that *g*_max_ differed significantly between males and females for *C. rumphii* [*F*_1,57_ = 5.44, *P*  = 0.0232] (Fig. 1B).

When examining the maximum respiration rate (*R*_max_), species was the only significantly predictor of *R*_max_ [species: *F*_4,57_ = 12.70, *P* < 0.0001; sex: *F*_1,57_ = 0.00, *P*  = 0.9988; sex × species: *F*_4,57_ = 0.42, *P*  = 0.7921]. Model contrast statements comparing the *R*_max_ of males and females within a species did not detect any significant differences (Fig. 1C).

### Leaf nutrient content

Total carbon content (%) differed significantly among species in this study [*F*_4,54_ = 10.22, *P* < 0.0001], with *Z. erosa* and *Z. portoricensis* containing the highest percentage [48.130 and 48.425, respectively]. Conversely, carbon content did not differ significantly between sexes [*F*_1,54_ = 0.65, *P*  = 0.4250], nor among the sex × species interactions [*F*_4,54_ = 1.04, *P*  = 0.3959]. Additionally, model contrast statements indicated no significant differences between male and female total carbon content within a species (data not shown).

**Figure 2:**
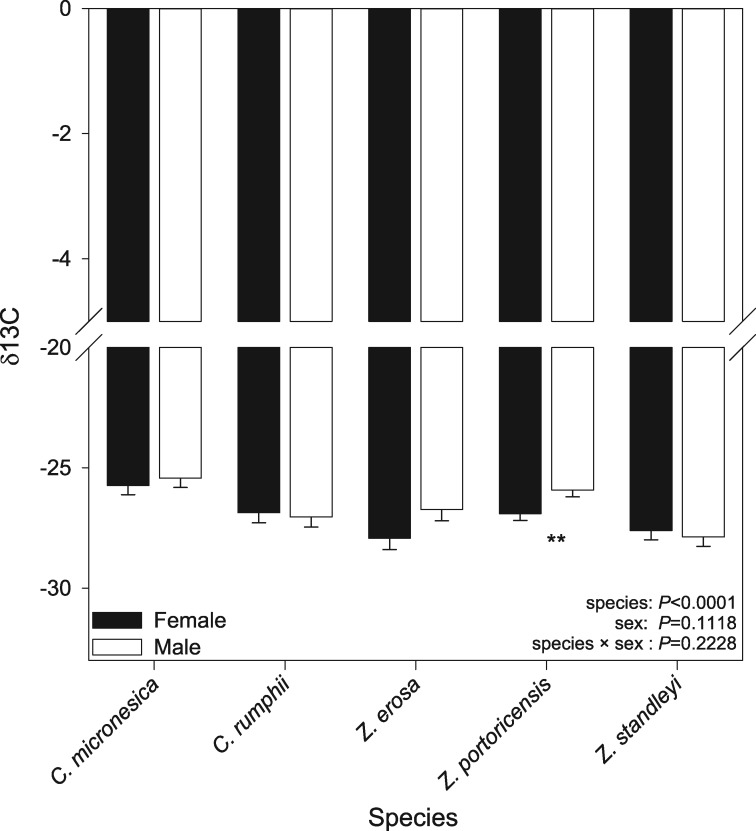
Mean (±1 S.E.) δ^13^C values for males (white bars), and females (black bars) in all species. Double asterisks indicate a significant difference (*P* < 0.05); error bars represent standard error of the mean.

With respect to δ^13^C, again species differ significantly [*F*_4,54_ = 9.39, *P* < 0.0001], with *Z. erosa* and *Z. standleyi* showing the most negative values [−27.331, −27.741, respectively]. Males and females did not differ in their discrimination of ^13^C overall [*F*_1,54_ = 2.61, *P*  = 0.1118], and the two way interaction between sex and species was not significant [*F*_4,54_ = 1.47, *P*  = 0.2228]. However, the model contrast statements show that males and females of *Z. portoricensis* differ significantly [*F*_1,54_ = 5.91, *P*  = 0.0184] (Fig. 2).

**Figure 3:**
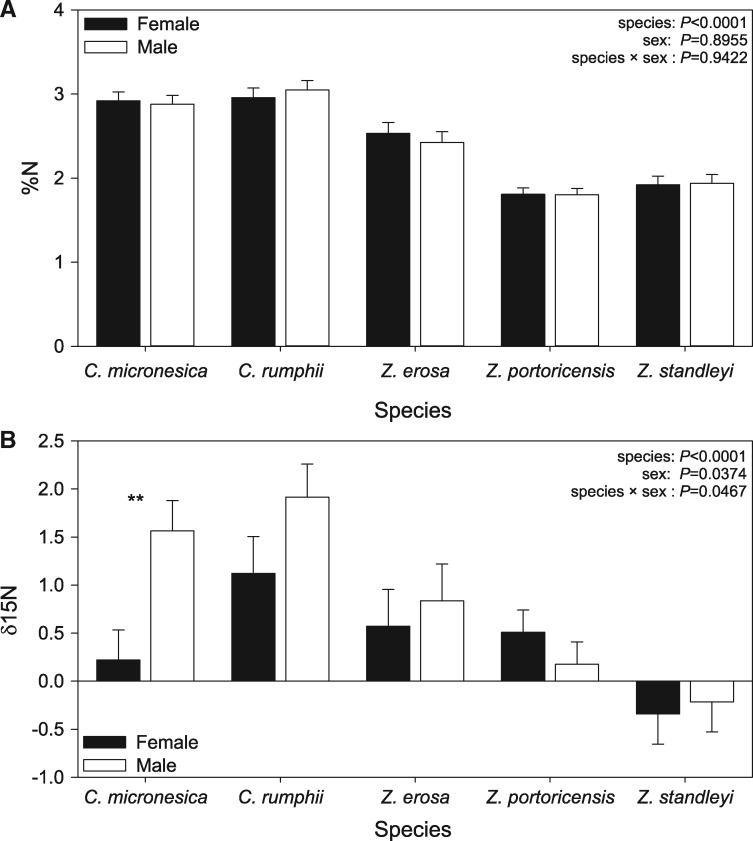
(A) Mean (±1 S.E.) N content (%), and (B) δ^15^N for males (white bars), and females (black bars) in all species. Double asterisks indicate a significant difference (*P* < 0.05); error bars represent standard error of the mean.

Nitrogen content (%) differed significantly among species [*F*_4,54_ = 63.31, *P* < 0.0001], but was insignificant for sex [*F*_1,54_ = 0.02, *P*  = 0.8955] and the subsequent two-way interaction [*F*_4,54_ = 0.19, *P*  = 0.9422]. For %N, *C. rumphii* and *C. micronesica* exhibited the highest levels of nitrogen. Males did not differ from females within a species as examined by model contrast statements (Fig. 3A).

Conversely, nitrogen isotope discrimination (δ^15^N) revealed a very different pattern, with sex [*F*_1,53_ = 4.56, *P*  = 0.0374], species [*F*_4,53_ = 8.12, *P* < 0.0001], and sex × species [*F*_4,53_ = 2.59, *P*  = 0.0467], being significant. Overall, males had a much greater δ^15^N [0.856] relative to females [0.416]. As with %N, *C. rumphii* and *C. micronesica* exhibited the highest levels of δ^15^N [1.52 and 0.89, respectively]. Contrast statements revealed a significant difference between males and females in *C. micronesica* [*F*_1,53_ = 9.22, *P*  = 0.0037], with males showing greater δ^15^N values than females (Fig. 3B).

### Stomata measurements

Species differed significantly in their stomatal densities [*F*_4,57_= 10.07, *P* < 0.0001], with *C. micronesica* having the greatest density (60.85 per cm^2^) and *Z. standleyi* showing the lowest density (38.08 per cm^2^). Both sex and the sex × species interaction were insignificant [sex: *F*_1,57_= 0.36, *P*  = 0.5529; sex × species: *F*_4,57_= 1.65, *P*  = 0.1744]. Model contrast statements indicated that males differed significantly from females in *C. micronesica* [*F*_1,57_ = 5.00, *P*  = 0.0293] with males having a greater stomatal density than females (66.96 and 54.74 per cm^2^, respectively) (data not shown).

## Discussion

Numerous studies have examined sex-specific resource utilization and the physiology of reproduction in dioecious flowering plants (e.g. Obeso 2002; Campbell 2000), and the majority of studies find females exhibit higher assimilate demand (Obeso 1997, 2002; Nicotra 1999a, 1999b; Banuelos and Obeso 2004; Cepeda-Cornejo and Dirzo 2010; Groen *et al.* 2010; Teitel *et al.* 2016). Such demands can be compensated by increased photosynthesis either on leaves near the site of reproduction, in foliar tissue in other parts of the plant, and/or by the reproductive organs themselves (Obeso 1997; Yu *et al.* 2013; Sunmonu *et al.* 2013). In cycads, both male and female plants make strobili, yet the female strobili must bear the developing ovule and seed. Female strobili are typically much larger than male strobili and in some species, these strobili represent a substantial component of the plant’s total biomass (Tang 1990). While not yet examined in cycads, increased photosynthetic rates that have been reported for female plants of other lineages can be dependent on developmental stage, with increases occurring during periods of flowering or fruiting (Reekie and Bazzaz 1987; Saulnier and Reekie 1995). For this reason, and in line with the finding of Clark and Clark (1988), we predicted that female reproduction would be more costly, and thus reproductive females would exhibit increased photosynthetic rates of the current season’s leaf cohort. Contrary to these predictions, photosynthetic rate was either the same in males and females or higher in males, although respiration rates did not differ between the sexes of any species. Whereas we were unable to directly assess reproductive costs, some studies have shown that male reproduction can be more costly (in terms of carbon) than female reproduction with males occasionally exhibiting increased photosynthetic rates (Nicotra *et al.* 2003; Gehring and Monson 1994).

Cycads are unusual in that the strobili of most species examined undergo periods of thermogenesis reaching temperatures in excess of 6 °C above ambient (Skubatz *et al.* 1993; Tang 1987; Tang *et al.* 1987). Although thermogenesis in cycads has not been extensively studied, male strobili often produce higher temperatures for longer periods of time than females, and female strobili of some species do not undergo thermogenesis (Tang *et al.* 1987; Seymour *et al.* 2004; Roemer *et al.* 2005; Suinyuy *et al.* 2010; Roemer *et al.* 2013; Terry *et al.* 2014). We still do not fully understand the link between resource allocation and thermogenesis, but this process likely represents a significant carbon cost for male plants (Tang *et al.* 1987), which may respond by increasing photosynthetic rates around periods of reproduction. Potential differences in thermogenesis may explain why some males showed elevated photosynthetic rates compared to females in some species, but not for others. The male strobili of all species examined likely undergo this process and it is possible that males only increase photosynthetic rates around the thermogenic period, which we may have missed in some species. Thermogenesis in cycads deserves immediate attention.

In some species, reproductive costs related to carbon can be offset by reproductive structures themselves. This has been shown for many seed plant taxa where flowering and fruiting structures can contribute directly to net carbon gain (reviewed in Raven and Griffiths 2015). In some ferns, however, the production of fertile fronds in monomorphic species comes at considerable costs and has long-term consequences for future reproduction (Britton and Watkins 2016; Watkins *et al.* 2016). Nothing is known of the photosynthetic capacity of microphylls and megaphylls in cycad strobili, yet most are green during early stages of development. In gymnosperms, female strobilus photosynthesis is often critical to strobilus formation, contributing a significant proportion of an individual’s respiratory costs, while male strobili seem to rely largely on foliar photosynthates for growth and development (McDowell *et al.* 2000; Matyssek and Benecke 2005; Wang *et al.*, 2006a, 2006b). If these structures in cycads contribute to overall carbon gain, this may explain the lack of difference found in photosynthetic and respiration rates between male and female leaves in some species.

Several studies have shown that physiological differences between males and females can influence the sex ratio within a population when environmental conditions are heterogeneous (Dawson and Bliss 1989; Dawson and Ehleringer 1993; Dawson and Geber 1999; Hultine *et al.* 2007) however only one study has examined this in cycads. Ornduff (1987) was the first to record that soil quality affects the sex ratio within populations of *Zamia pumila.* He found in shallow, poor soils, populations were male biased, and females reproduced less frequently. In sites with more favorable soils, the proportion of males and females was equal, and females reproduced more frequently. Ornduff noted the importance of soil depth because of the direct relationship to water availability. Although water availability is a critical component of the plant-soil interface, studies of experimental manipulation of any abiotic factor are lacking from the cycad literature. Heterogeneity in water availability of soils can influence the spatial distribution of other dioecious plant species and influence sex ratios within natural populations (Freeman *et al.* 1976; Houssard *et al.* 1992; Correia and Barradas 2000; Dudley 2006; Xu *et al.* 2008). However, individuals included in this study were subjected to a common water-regime which minimized the effect of differing water-availability. Furthermore, all species were grown at the same botanical garden and thus share many common environmental conditions. Because all species were subjected to a common water-regime, we did not expect to see differences in water-use unless such differences were truly sex-specific. Despite males generally having higher photosynthetic rates, in some species females showed greater water-use efficiency (more negative δ^13^C).

Reproductive costs are not only related to carbon, as nitrogen plays a key role in most physiological processes and some studies have shown that males and females can have different nitrogen costs or that nitrogen content varies over the reproductive period (e.g. Ågren 1988; Barradas and Correia 1999; Harris and Pannell 2008; Van Drunen and Dorken 2012; Sánchez-Vilas and Retuerto 2016). All cycad species produce colloroid roots that become infected with nitrogen fixing cyanobacteria (Milindasuta 1975; Whitelock 2002). The role that these symbiotic cyanobacteria play in resource allocation in cycads has received little attention, yet some studies have shown that symbiotic cyanobacteria contribute directly to the nitrogen budget of the above and belowground tissues (Halliday and Pate 1976; Lindblad 1990; Rai *et al.* 2000). Nitrogen fixing soil bacteria discriminate between naturally occurring ^15^N and ^14^N which results in enriched ^15^N signatures (Handley and Raven 1992; Wanek and Ardnt 2002; Dawson *et al.* 2002; Reich *et al.* 2003; Watkins *et al.* 2007; Watkins *et al.* 2008; Spriggs and Dakora 2009; Schweiger *et al.* 2014). We found marked differences of δ^15^N among species, as well as between male and females. Males were generally more enriched relative to females in the *Cycas* species and in most *Zamia* species. This pattern was most striking in *Cycas micronesica* and suggests that females rely more heavily on N derived from cyanobacteria than males. Surprisingly, little is known regarding the variation of ^15^N in dioecious plants (but see recent work by Sánchez-Vilas and Retuerto 2016). Even less is known of the mechanisms of how different sexes can alter cyanobacterial infection, or how the costs and pay-offs of this symbiosis may differ. However, recent studies have shown that other root associations, such as with mycorrhiza, can differ between sexes in dioecious plant species, and that this association can also disproportionately benefit one sex (Varga and KytövIIta 2008; Vega-Frutis and Guevara 2009; Varga and Kytöviita 2010; Varga *et al.* 2013). 

## Conclusions

We found several patterns of physiological and morphological differences between sexes that provide novel research areas in need of further exploration. Of particular interest is the symbiotic relationship between nitrogen-fixing cyanobacteria and the colloroid roots of cycads. Little is known about this relationship, and our results indicate the nitrogen benefits of these symbiotic relationships may differ between males and females. Nitrogen fixing soil bacteria have been co-evolving with cycads for more than 300 million years (Vessey *et al.* 2005) and may be an important factor in maintaining stable dioecy in some species of this ancient lineage.

Many other aspects of cycad biology remain poorly understood, including the period of thermogenesis in male strobili. We know little about the role thermogenesis plays in the resource budget of cycads. Fortunately, recent studies have sparked a resurgence of interest in cycad biology; however, cycads are still understudied despite representing a key phylogenetic position among extant vascular land plants as the first seed-bearing dioecious lineage, and the only gymnosperms that form symbiotic relationships with N-fixing bacteria in their roots. Studies of cycads will dramatically improve our understanding of plant evolution and studies that focus on the above topics are desperately needed in the scientific literature.

## Sources of Funding

Our work was funded by the Colgate Research Council, Colgate University.

## Contributions by the Authors

C.K. and J.E.W. conceived of the research project; C.K. collected data; C.K., J.E.W., S.C., and C.H., analysed data and wrote the manuscript.

## Conflict of Interest Statement

None declared.
